# Effects of Pre-Parturient Iodine Teat Dip Applications on Modulating Aversive Behaviors and Mastitis in Primiparous Cows

**DOI:** 10.3390/ani11061623

**Published:** 2021-05-31

**Authors:** Hannah N. Phillips, Ulrike S. Sorge, Bradley J. Heins

**Affiliations:** 1 Department of Animal Science, University of Minnesota, Saint Paul, MN 55108, USA; 2 Department of Veterinary Population Medicine, University of Minnesota, Saint Paul, MN 55108, USA; ulrike.sorge@tgd-bayern.de; 3 Bavarian Animal Health Services, 85586 Poing, Germany

**Keywords:** human–animal relationship, training, acclimating, heifer, primiparous, dairy, behavior, welfare, udder health, mastitis

## Abstract

**Simple Summary:**

The first few days after calving can be stressful for young dairy cows since they must acclimate to many unfamiliar situations that are required for milking, including novel noises and sensations to their udders. Furthermore, the human–animal relationship is simultaneously jeopardized during this time since cows may display undesirable or dangerous behaviors during milking, such as kicking and stomping. These cows are also at risk for bacterial mammary infections that can be painful and damage tissue. In a controlled experiment, young cows that investigated the milking area and had their teats sanitized weekly for 3 weeks prior to calving were more comfortable during post-calving milking procedures as indicated by reduced kicking and restlessness behaviors. Furthermore, they had fewer mammary infections caused by the bacteria *Staphylococcus aureus* after calving. Results from this experiment indicate that sanitizing teats and providing opportunities to acclimate to the milking area prior to calving may improve the wellbeing and mammary health of young cows and promote a positive human–animal relationship.

**Abstract:**

Heifers and their human handlers are at risk for decreased welfare during the early lactation period. This experiment investigated pre-parturient teat dipping and parlor acclimation to reduce mastitis and aversive behaviors in early lactation heifers. Three weeks prior to calving, heifers were randomly assigned to receive either: (1) a weekly 1.0% iodine-based teat dip in the parlor (trained; *n* = 37) or (2) no treatment (control; *n* = 30). For the first 3 days of lactation, heifers were milked twice daily, and treatment-blinded handlers assessed behaviors and clinical mastitis. Aseptic quarter milk samples were collected within 36 h of calving and analyzed for pathogens. Control heifers had (OR ± SE) 2.2 ± 0.6 times greater (*p* < 0.01) odds of kicking during milking. Trained heifers had (OR ± SE) 1.7 ± 0.4 times greater (*p* = 0.02) odds of being very calm during milking, while control heifers had 2.2 ± 0.8 and 3.8 ± 2.1 times greater (*p* < 0.04) odds of being restless and very restless or hostile during milking, respectively. Quarters of control heifers had (OR ± SE) 5.4 ± 3.4 greater (*p* < 0.01) odds of intramammary *Staphylococcus aureus* infection, yet clinical mastitis was similar among treatments. The results indicate that teat dipping in the parlor weekly for 3 weeks before calving may alleviate some aversive milking behaviors and protect against early lactation *S. aureus* intramammary infections.

## 1. Introduction

The periparturient period is particularly challenging for heifers. For one, the new experience of being milked may be distressing for some heifers. This may not only jeopardize animal welfare, but also endanger the safety of human handlers, as distressed heifers may kick off milking clusters, kick at handlers and display other aversive behaviors that interfere with milking efficiency. This increases the chance of injury to handlers and the risk of mastitis for the animal [1,2,3,4]. In addition, heifers are susceptible to intramammary infections (IMIs) and clinical mastitis during this time [5,6,7]. While late gestation IMI and early lactation mastitis are associated with an economic loss due to treatment costs [8], production loss [9,10], risk of future infections [11,12], reduced reproductive performance [13] and increased risk of culling [14,15], negative affective states and poor milking behavior may also represent an economic loss due to risk of IMI [1], decreased milk productivity [16] and the risk of early culling [3]. Therefore, preventing early lactation distress and mastitis in heifers may be of interest in terms of improving animal welfare and economic benefit.

Several strategies have been studied to reduce the behavioral reactivity of heifers and improve the human–animal relationship. These include the positive handling of heifers and familiarizing them with the milking parlor before calving [3,17,18,19,20,21,22]. For example, the positive handling of heifers during the calving process reduced the number of flinch, step and kick responses performed during milking procedures over the first 20 weeks of lactation [3]. Bertenshaw et al. [18] found that spending 30 to 245 min brushing heifers during the last 6 weeks of gestation can reduce kicking behaviors during milking procedures up to 4 weeks after parturition. Kutzer et al. [20] showed that pre-parturient training, which consisted of the introduction to the milking herd at least 10 days before calving and at least 10 visits to the parlor where milking staff provided tactile contact to the udder on the milking platform, reduced post-parturient aversive behaviors in heifers. However, intensive habituation protocols such as the ones described in previous experiments may not be feasible for many farms due to labor and infrastructure restraints, so it is important to devise a simpler habituation protocol that fits within the capabilities of dairy farms.

Protocols to prevent IMI and clinical mastitis have been examined using a variety of pre-parturient techniques, such as internal teat sealants [6,23,24,25], antibiotic therapies [26,27,28,29,30], milking [22,31,32] and repeated applications of teat dip or spray [14,33,34]. However, many of these methods, including internal teat sealants and antibiotics, are not allowed for use in organic dairy animals in the United States. Furthermore, labor limitations prevent the implementation of intensive protocols on most farms, especially those that house heifers on pasture. Therefore, the current protocol for preventing udder health issues in pre-parturient heifers needs modification to be feasible.

The objectives of this study were to determine whether weekly pre-parturient teat dipping in the milking parlor would modulate behavioral responses and decrease the mastitis and IMI risk of heifers over the first 3 days of the lactation period.

## 2. Materials and Methods

### 2.1. Animals and Housing

The University of Minnesota Institutional Animal Care and Use Committee approved all animal care and procedures specific to this experiment (protocol number #1906-37134A).

The experiment was conducted from March to November 2018 at the University of Minnesota West Central Research and Outreach Center (Morris, MN) using heifers in conventional and organic dairy research herds. Details on calf housing and care are described in Kienitz et al. [35]. Heifers used in this study were either pure Holstein or one of two crossbreeds, as described by Heins et al. [36].

Heifers were housed on pasture from 6 months of age and were supplemented with a total mixed ration during the nongrazing season. Heifers were first artificially inseminated after reaching 14 months of age during the winter (December to February) and summer (June to August) breeding seasons for the subsequent fall and spring calving seasons, respectively. Heifers were culled from the herd if they did not become pregnant after 2 breeding seasons.

Pregnant heifers were moved to a maternity pen 4 weeks prior to their calving due date. The range for age at calving was 691 to 791 days (mean ± SD = 726 ± 20 days). Heifers were housed in a compost-bedded pack barn and an outdoor straw pack for 24 h after calving for health monitoring. Details on theses housing systems are described by Sjostrom et al. [37]. Twenty-four hours after calving, heifers moved into their respective conventional or organic lactating herd. The conventional or organic status of the heifer was based on the status of the heifer’s dam.

Details on the conventional and organic lactating herd housing and feeding management are described by Minegishi et al. [38]. In brief, the organic herd was housed on pasture from May to October, where they had ad libitum access to forages for grazing, water and minerals and were fed 2.72 kg per head of organic corn daily. From November to April, the organic herd was housed in a compost-bedded pack barn or outdoor straw pack, where they were fed an organic total mixed ration. Meanwhile, the conventional herd was housed in an uncovered dry-lot from May to October and in a compost-bedded pack barn or outdoor straw pack from November to April. The conventional herd was fed a conventional total mixed ration.

Heifers were milked twice daily at 06:00 and 17:00 h in a swing-nine parabone milking parlor after calving. At each milking, heifers were pre- and post-dipped with a 1.0% iodine-based teat dip. Teats were not dried before heifers exited the milking parlor. If the ambient outdoor temperature was less than −10 °C, heifers were post-dipped with an organically approved restricted-use chlorhexidine powder teat dip to prevent frostbite.

### 2.2. Experimental Design

This study used a generalized randomized complete block design where the season (spring and fall) served as the blocking factor. Before calving, heifers were allocated randomly to one of two treatment groups: (1) trained (*n* = 37) or (2) control (*n* = 30). Treatments were balanced for calving date. The number of animals per treatment according to breed, season and herd is listed in Table 1. Heifers allocated to the training treatment received a training program in the swing-nine milking parlor once weekly for 3 consecutive weeks until calving, whereas those in the control treatment remained in their home pen.

Training began 4 weeks prior to the expected calving date of heifers. On each Tuesday between 12:00 and 14:00 h, heifers to be trained were brought to the parlor in groups of 6 to 9, wherein they could investigate the holding pen for 15 min. Then, they were loaded into the milking parlor by a handler using gentle stockmanship practices, which included quiet voice prompts, gentle hand and arm movements and light tactile force with hands if necessary. The parlor holding gate was then brought down to secure heifers in place to simulate normal milking. Then, each teat was cleaned with a single use towel and dipped with a 1.0% iodine-based barrier teat dip (Chem-Star Io-Soft 1000 + 10, Ecolab, St. Paul, MN, USA) by a handler. Heifers remained in the parlor for 5 min until they were released to return to their home pen. Trained heifers received either 3 or 4 training sessions based on whether they calved near or before their expected calving date.

### 2.3. Data Collection

After calving, heifers were observed during each milking for 3 days. Treatment-blinded farm staff scored heifers according to similar methods described by Tulloh [39] for parlor entry ease (1 = willing; 2 = willing but hesitates; 3 = requires crowd gate prompt; 4 = requires handler to enter holding pen) and milking ease (1 = very calm; 2 = calm; 3 = restless; 4 = very restless; 5 = hostile). While heifers were in the parlor, farm staff recorded whether the following behaviors were performed: stomp, kick, defecate and kick off milker. An ethogram for these behaviors is provided in Table 2. Farm staff also assessed heifers for udder edema and clinical mastitis at each milking. Udder edema was defined as a swollen and firmer udder base without abnormal milk. Clinical mastitis was scored by visual observation of stripped milk from each quarter as follows [40]: normal (score 0) = milk is normal; mild (score 1) = milk contained flakes, clots or serum; moderate (score 2) = mild mastitis accompanied by swelling or redness of the mammary gland or quarter; or severe (score 3) = moderate mastitis accompanied by systemic signs of illness such as depression, anorexia, dehydration and/or fever. The 6 farm staff workers who observed and recorded data had at least 90% agreement on all recorded outcomes with the principal investigator of the study, which was assessed at the beginning of the fall and spring calving seasons.

After colostrum was collected, aseptic quarter milk samples were collected within 36 h of calving and analyzed for pathogens at the University of Minnesota Veterinary Diagnostic Laboratory (St. Paul, MN, USA). Before collection, loose manure, dirt and bedding particles were brushed off from the udder and teats, and teats were dipped with a 1.0% iodine pre-dip. Thirty seconds after the dip was applied, teats were wiped clean with a single-use paper towel and the first 3 to 4 streams of milk from each teat were discarded. The apex of each teat was repeatedly scrubbed with a new gauze pad soaked in 70% isopropyl alcohol until a withdrawn pad was unsoiled. Milk (20 to 30 mL) was collected in sterile plastic tubes. During the sampling process, disposable gloves were changed between heifers. Samples were frozen within 24 h of collection and were later shipped overnight with frozen gel packs to the lab for analysis within a month of collection.

For each quarter sample, a volume of 0.1 mL of milk was swabbed onto each section of a half-plate containing either Factor media (Gram-positive selective; University of Minnesota, St. Paul, MN, USA) or MacConkey II (Gram-negative selective; Becton Dickinson, Franklin Lakes, NJ, USA). The plates were incubated at 37 °C for 48 h. After 24 h, the plates were examined for growth and identification procedures were performed. After 48 h, the plates were re-examined for growth and identification if new growth was present. For each sample, the level of bacteria growth was assessed and categorized as either no (<1 CFU/0.1 mL), low (1 to 10 CFU/0.1 mL), medium (11 to 50 CFU/0.1 mL) or high growth (>50 CFU/0.1 mL). A sample was considered contaminated if more than 2 types of bacteria were present. Contaminated samples were not evaluated for bacteria species. For plates with growth, the colonies were differentiated by using matrix-assisted laser desorption/ionization time-of-flight mass spectrometry (MALDI Biotyper, Bruker, Billerica, MA, USA) [41]. Identification was performed to the species level when the CI was at least 2.00, and to the genus level if the CI was 1.80 to 1.99. 

Pathogens were categorized according to Passchyn [42]. *Staphylococcus (S.) chromogenes*, *Staphylococcus sciuri*, *Staphylococcus hominis*, and other non-*aureus* (and coagulase negative) *Staphylococcus* spp. were combined into a single category of coagulase-negative staphylococci (CNS) [43]. The major contagious pathogen was *Staphylococcus aureus*. The other major pathogens were *Streptococcus (S.) dysgalactiae*, *Streptococcus uberis*, *Enterobacter (E.) cloacae* and *Escherichia (E.) coli*. Coagulase-negative staphylococci, non-*agalactiae Streptococcus* spp., *Bacillus* spp., *Enterococcus (E.) faecalis*, *Serratia* spp. and unidentified Gram-positive cocci were the minor pathogens. Twenty-nine quarter samples had 2 species of bacteria identified, so only 1 species was kept for subsequent analyses, as described by Parker et al. [6]. When there was a mixed major and minor pathogen infection present (8 quarters), the quarter was defined as being infected with the major pathogen. In 1 quarter, there were 2 major pathogens isolated (*S. aureus* and *S. uberis*), so *S. aureus* was the species used in further analysis. When there were 2 minor pathogens present at differing levels (11 quarters), the quarter was defined as being infected with the pathogen of greater CFU in subsequent analyses. In 9 quarters, 2 minor pathogens at the same level were isolated (CNS and *Bacillus* spp. in 7 quarters; CNS and Gram-positive cocci in 1 quarter; and *Serratia* spp. and *Bacillus* spp. in 1 quarter), so CNS or *Serratia* spp. were the species used in further analysis.

### 2.4. Data Analysis

Data collected during parlor visits were aggregated across milkings. Similarly, udder health data from milk samples were aggregated across quarters. Some of the outcome variables were coerced into categorizes for the analysis. Entry ease scores 3 and 4 (score > 2) and milking ease scores 4 and 5 (score > 3) were combined into single score categories. Clinical mastitis and udder edema data were dichotomized into either absent during all milkings or present during at least 1 milking over the 3-day observation period. *S. dysgalactiae*, *S. uberis*, other non-*agalactiae Streptococcus* spp., *Bacillus* spp., *E. cloacae*, *E. faecalis*, *E. coli*, *Serratia* spp. and unidentified Gram-positive cocci were categorized as other environmental pathogens. Heifers with all quarter samples contaminated (10 heifers) were removed from the quarter milk analyses. Furthermore, seven and 63 quarter samples were excluded from the analysis due to being either missing or contaminated, respectively [44]. Therefore, 159 quarters were evaluated.

Analyses were performed in RStudio (version 1.3.1103) [45] with logistic regression using the *glm* function. The fixed effects were age at calving (continuous), season (2 levels), breed (3 levels), herd (2 levels) and treatment (2 levels). For the analyses of behaviors, the fixed effect of clinical mastitis (2 levels) was included in the models. Likelihood ratio tests (LRTs) were used to assess the significance of effects by comparing full and reduced models [46]. Significance was declared when *p* < 0.05. Marginal means and CI for behaviors are reported as probability values back-transformed from the logit scale. Odds ratios (ORs) and SE are used to compare treatment groups.

## 3. Results

### 3.1. Behaviors

Treatments were different for kicks and milking ease, while stomping, defecating, kicking the milking unit off, and entry ease were similar between treatments (Table 3). In general, trained heifers had preferred behaviors even after accounting for clinical mastitis. Control heifers had (OR ± SE) 2.2 ± 0.6 times greater odds of kicking during milking procedures compared to trained heifers. Trained heifers had (OR ± SE) 1.7 ± 0.4 times greater odds of being very calm during milking (milking ease score = 1) compared to control heifers, while control heifers had 2.2 ± 0.8 times greater odds of being restless during milking (milking ease score = 3) and 3.8 ± 2.1 times greater odds of being very restless or hostile during milking (milking ease score > 3) compared to trained heifers. There was an effect of calving age on stomping (*χ*^2^_(1)_ = 5.3, *p* = 0.02), kicking (*χ*^2^_(1)_ = 2.2, *p* = 0.03) and kicking off the milking unit (*χ*^2^_(1)_ = 8.2, *p* < 0.01), in which older heifers were more likely to perform this behavior.

Clinical mastitis influenced some behaviors, in which heifers with clinical mastitis were more likely to hesitate while entering the parlor, kick during milking, and be very restless or hostile during milking compared to heifers without clinical mastitis. Heifers with clinical mastitis had (OR ± SE) 2.3 ± 0.7 times greater odds of kicking (*χ*^2^_(1)_ = 7.7, *p* < 0.01), 3.0 ± 1.0 times greater odds of hesitantly entering the parlor (entry ease score = 2) (*χ*^2^_(1)_ = 11.6, *p* < 0.01) and 2.7 ± 1.4 times greater odds of being very restless or hostile during milking (milking ease score > 3) (*χ*^2^_(1)_ = 3.9, *p* = 0.048) compared to heifers without clinical mastitis. Meanwhile, heifers without clinical mastitis had (OR ± SE) 3.0 ± 0.8 times greater odds of entering the parlor willingly (entry ease score = 1) (*χ*^2^_(1)_ = 17.4, *p* < 0.01) compared to heifers with clinical mastitis.

### 3.2. Udder Health

Clinical mastitis and udder edema results are in presented in Table 3. All observed clinical mastitis cases were either mild (score 1) or moderate (score 2). Clinical mastitis was recorded at 13.9% of milkings on average. Over the first 3 d of lactation, approximately one-third (32.8%) of heifers developed signs of clinical mastitis and 13.4% had signs of udder edema. There was an effect of age on clinical mastitis, such that the odds of showing signs of clinical mastitis during the first 3 d of lactation decreased as calving age increased (*χ*^2^_(1)_ = 8.4, *p* < 0.01).

There was an effect of treatment on quarter IMI and pathogens isolated (Table 4). Control heifer quarters had (OR ± SE) 5.4 ± 3.4 greater odds of being infected with *S. aureus*. Over a one-third (37.1%) of quarters and 15.8% of heifers were pathogen-free. *S. aureus* was the only contagious bacteria found in 9.4% of quarters. On average, the most common pathogen found was CNS, which was identified in 62 (39.0%) of quarters (*S. chromogenes* (22.0% of quarters), other non-*aureus Staphylococcus* spp. (15.1% of quarters), *S. sciuri* (1.3% of quarters) and *S. hominis* (0.6% of quarters)). Other pathogens were identified in 23 (14.5%) of quarters (*Bacillus* spp. (6.9% of quarters), *S. dysgalactiae* (1.9% of quarters), unidentified Gram-positive cocci (1.3% of quarters), *S. uberis* (1.3% of quarters), *E. cloacae* (0.6% of quarters), *E. faecalis* (0.6% of quarters), *E. coli* (0.6% of quarters), other non-*agalactiae Streptococcus* spp. (0.6% of quarters) and *Serratia* spp. (0.6% of quarters)).

Herd and breed influenced some udder health outcomes. Quarters of conventional heifers had a greater probability of having no growth compared to organic (50.0 vs. 30.6%; *χ*^2^_(1)_ = 4.3, *p* = 0.04). Quarters of organic heifers had a greater probability of being pathogen-positive at a high level compared to conventional (24.3 vs. 5.7%; *χ*^2^_(1)_ = 10.3, *p* < 0.01). There was an effect of breed on quarter infection level, in which Holstein quarters had greater (*p* = 0.03) odds of high CFU growth compared to Normande, Jersey and Viking Red crossbred quarters, while Montbéliarde, Viking Red and Holstein crossbred quarters had similar (*p* ≥ 0.20) odds of high CFU growth compared to Normande, Jersey and Viking Red crossbred and Holstein quarters (*χ*^2^_(2)_ = 7.6, *p* = 0.02).

## 4. Discussion

### 4.1. Behaviors

Trained heifers were less likely to kick and were more likely to be very calm during milking procedures, while control heifers were more likely to be very restless or hostile during milking procedures. These results indicate that the training program modulated aversive milking procedure behaviors to some extent. However, there were no effects of treatment on behaviors related to stomping, defecating, kicking off the milking unit and parlor entry ease. In a similar experiment, buffalo heifers that were habituated daily to milking parlor procedures for approximately 13 consecutive days before their expected calving date showed a reduction in the number of steps and kicks up to 20 d after calving [47]. Kutzer et al. [20] showed that pre-parturient training, which consisted of the introduction to the milking herd at least 10 days before calving and at least 10 visits to the parlor where milking staff provided tactile contact to the udder on the milking platform, reduced aversive behaviors in post-parturient heifers, such as stepping and kicking during milking procedures and tail clamping while entering the parlor. Das and Das [21] showed that at least 30 udder massage sessions 2 months prior to calving improved heifer temperament and reduced defecating during milking procedures on the first day of the lactation period. These previous experiments utilized a more rigorous training program than the present experiment, which employed weekly training for the 3 weeks leading up to parturition. Therefore, it is possible that the training regimen used in the present experiment was only sufficient in reducing kicks and improving milking procedure ease. Nevertheless, the training program used for the present experiment successfully modulated some aversive behaviors in heifers.

The training program used in this experiment represents a practical option for farmers that balances between modest and intensive training programs. For example, Ivemeyer et al. [48] reported heifers that received 4 handling sessions consisting of light touching on the neck by handlers 2 times per day on 2 separate days beginning 11 days before calving showed no difference in step or kick behaviors compared to non-handled heifers. On the contrary, intensive training programs requiring daily sessions consisting of passing through the milking parlor and receiving tactile contact at the udder, including washing, massaging and teat stripping, over approximately 13 to 14 days prior to parturition successfully improved heifer aversive behaviors [20,47]. Even though the training program used for the present experiment only required 3 sessions, it was sustained over a longer duration than described in previous experiments, which may explain why it successfully improved some aversive behaviors. For example, Boissy and Bouissou [49] suggested that additional handling may reduce the reactivity of heifers to humans when the handling program is sustained over a longer period of time (30 times spread out over 10 months) compared to intensive handling over a short period of time (30 times spread out over 3 months).

### 4.2. Udder Health

#### 4.2.1. Clinical Mastitis

Contrary to our hypothesis, the development of clinical mastitis during the first 3 days of lactation was similar between treatments. This finding is similar to previous experiments that reported that iodine-based teat spraying or dipping of heifers during the last weeks before calving does not reduce early lactation clinical mastitis [33,50]. Approximately one-third of heifers showed signs of clinical mastitis over the first 3 days of lactation, which is similar to previous experiments, which reported that 22 to 23% of pasture-based heifers were diagnosed with clinical mastitis over the first 2 weeks of lactation [6,44]. Similarly, another experiment reported that 28% of the heifers developed signs of clinical mastitis during the first 5 days of lactation [50].

#### 4.2.2. Quarter-Level Udder Health

Quarters of trained heifers were less likely to be infected with *S. aureus*, but overall intramammary infection levels were similar between treatments. These results suggest that the training regimen implemented for this experiment successfully reduced quarter-level *S. aureus* IMI. This finding is in agreement with previous experiments, which showed that spraying or dipping heifer teats during the last weeks before calving can reduce the prevalence of certain IMIs at calving [33,34]. For example, Lopez-Benavides et al. [33] reported that thrice-weekly application of iodine-based teat spray 3 weeks prior to calving reduced *S. uberis* but not CNS IMI. In contrast, Edinger et al. [50] found that teat dipping with 0.1% povidone iodine thrice-weekly for 3 weeks prior to calving did not reduce incidences of *S. aureus* or CNS IMI for heifers up to 5 days in milk. The distribution and types of pathogens found in infected quarters is in agreement with results from previous experiments, which also reported that CNS was the most common bacteria isolated from post-parturient heifers [6,25,27,30,50,51,52,53].

#### 4.2.3. Significance of *S. aureus*

Early lactation *S. aureus* IMIs are more likely to persist throughout lactation compared to other pathogen-specific IMIs and contribute to milk yield losses and increased somatic cell counts [13,54], proving to be one of the most difficult and important pathogens to control [55]. The estimated median duration of subclinical *S. aureus* infections is 64 and 91 days [56], and the likelihood of bacteriological cure is low [57]. Although *S. aureus* is generally lower in heifers compared to multiparous cows during early lactation [58], it can still be a major cause of clinical mastitis in heifers [7,59]. *S. aureus* is a contagious pathogen that is predominantly transmitted between herd mates [60] and quarters of the same cow [12]. However, infectious genotypes of *S. aureus* found in milk are also observed on the bodies of animals (e.g., skin and mucosal membranes) and in their environment [61,62].

In the present experiment, teat dipping heifers weekly 3 weeks before calving reduced quarter-level *S. aureus* IMI identified immediately after the termination of colostrum (within 36 h after calving). The mechanism of action that pre-parturient teat dipping had on the reduction in post-parturient *S. aureus* IMI may be described by a variety of plausible explanations. It is possible that the teat dip prevented new *S. aureus* IMI during the treatment period. Up to 15% of quarters may be infected with *S. aureus* 1 to 3 weeks prior to calving [27,28,29,44,52,63]. Iodine teat dips at 0.1 to 0.5% have been shown to effectively prevent *S. aureus* IMI in lactating cows by 63.3 to 88.2% [64,65,66], so it is possible that that teat dip killed *S. aureus* on the teat end and prevented new *S. aureus* IMIs in pre-parturient heifers.

#### 4.2.4. IMI Rate of Research Population

It appears that the population of heifers used for the present study had a higher rate of IMI than previous studies. For example, previous experiments reported that 48 to 91% of quarter samples taken within 5 days after calving had negative bacteriological culture results, which is greater than the 37% found for the present experiment [1,5,6,25,44,50,51]. Bludau [5] reported that 25% of heifers were non-infected 24 h after calving, which is greater than the 16% found in the present experiment. Furthermore, the presence of *S. aureus* (9.4% of quarters) and CNS (39.0% of quarters) in quarters for the present experiment are much higher than many previously reported values, in which previous experiments reported that approximately 0.6 to 2.7% and 4.8 to 9.7% of quarters were infected with *S. aureus* and CNS within 5 days after calving, respectively [1,6,32,44]. On the contrary, a few experiments reported comparable intramammary infection rates within 48 h after calving for *S. aureus* (3.9 to 10.9% of quarters) and CNS (33.2 to 47.3% of quarters) [53,67]. It is possible that herd-level specifics and farm management contributed to the high level of IMI in the population of heifers used in the present experiment.

The high level of IMI found in the present experiment may be greater than previous reports due to atypical housing and management, such as pasture housing and a lack of antibiotic therapies. For example, pastured heifers may have had an increased risk of *S. aureus* IMI due to biting flies transferring the pathogen between infected and non-infected animals [68]. Based on previous experiments using the same research herd [69], it is possible that stable flies (*Stomoxys calcitrans* L.) and horn flies (*Haematobia irritans* L.) may be a contributing factor to the spread of *S. aureus* within the herd. Furthermore, the outdoor housing used in the current experiment could have increased the risk of elevated IMI, mainly due to unhygienic legs and udders caused by heavy precipitation events followed by wet and muddy surroundings [44,70].

#### 4.2.5. Other Effects on Udder Health

Pathogen-positive quarters at a high CFU level were more commonly observed in quarters of organic heifers and of Holstein heifers. Similarly, Persson Waller et al. [14] reported lower somatic cell counts in conventional heifers compared to organic heifers over the first 2 milkings of the lactation period. For the present experiment, it is possible that differences between conventional and organic post-parturient housing and genetic differences played a major role in the development of IMI and clinical mastitis. Previous experiments reported that unhygienic legs and udders may put cows at risk for clinical and subclinical mastitis due to environmental pathogens [44,70]. Furthermore, Persson Waller et al. [7] also reported a possible effect of breed on udder health, indicating that breed may play a role in susceptibility to mastitis.

### 4.3. Limitations

Although the training program was associated with positive effects on animal behavior and udder health in the present experiment, the limited number of animals and fairly high incidence of clinical mastitis will need to be considered before generalization of the results. Producers should consider how differences in animals, management and environment could play a role in the application of these results. For example, heifer temperament [2,71] and milking parlor type [72] may influence the behavioral outcomes of this training protocol. Likewise, herd-specific factors, such as the current level of IMI, may influence outcomes of teat dipping pre-parturient heifers, such that improvements may be unapparent or more apparent if certain pathogens are present or absent [58]. Therefore, future investigations of aversive behavior and mastitis prevention strategies using methods such as those of the present experiment should be investigated under a variety of management systems.

## 5. Conclusions

Teat dipping pre-parturient heifers in the milking parlor weekly beginning 3 weeks before calving reduced the occurrence of some aversive behaviors and the risk of *S. aureus* IMI in early lactation heifers. Such treatment led to improved milking ease scores and reduced kicks during milking procedures over the first 3 days of the lactation period. However, pre-parturient treatment was not associated with significantly improved parlor entry ease scores nor reduced stomping, defecating or kicking off milking units. The pre-parturient treatment resulted in reduced *S. aureus* IMI observed immediately after the collection of colostrum (within 36 h of calving). Yet, the development of clinical mastitis and udder edema in heifers over the first 3 days of the lactation period was not affected by pre-parturient treatment. Likewise, pre-parturient treatment did not markedly reduce overall quarter IMI and resulted in comparable quarter IMI caused by CNS and other environmentally transmitted pathogens. Therefore, the results from this experiment suggest that weekly teat dipping 3 weeks before the expected calving date may modulate some aversive behaviors and *S. aureus* IMI in early lactation heifers.

## Figures and Tables

**Table 1:** Distribution of animals by treatment and group.

	Treatment	
Group	Trained (*n* = 37)	Control (*n* = 30)
Breed ^1^		
Holstein	11	9
MVH	16	18
NJV	10	3
Season		
Fall	25	21
Spring	12	9
Herd		
Conventional	12	21
Organic	25	9

^1^ MVH = Montbéliarde, Viking Red and Holstein crossbred; NJV = Normande, Jersey and Viking Red crossbred.

**Table 2:** Ethogram of behaviors recorded during milking adapted from Eicher et al. [22].

Behavior	Description
Stomp	A raising and lowering of the foot in one place
Kick	A forward or sideways leg movement, without dislodging claws
Defecate	A discharge of feces
Kick off milker	A kick that causes the claws to fully or partially dislodge

**Table 3:** The probabilities (95% CI) of behaviors occurring during milkings and of the development of clinical mastitis and udder edema over the first 3 d of lactation in heifers receiving a weekly 1.0% iodine-based teat dip in the parlor 3 weeks prior to calving (trained) or no treatment (control) based on logistic regression.

	Treatment		Treatment Effect ^1^		Odds Ratio (OR) ± SE
Behaviors and Udder Health, % Probability	Train (*n* = 37)	Control (*n* = 30)	*χ* ^2^_(1)_	*p*-Value	Train vs. Control
Stomp	62.0 (54.1, 69.3)	65.4 (56.6, 73.2)	0.4	0.53	0.9 ± 0.2
Kick	16.5 (11.4, 23.2)	29.8 (22.1, 38.9)	7.0	<0.01	0.5 ± 0.1
Defecate	9.6 (5.9, 15.3)	12.0 (7.3, 19.1)	0.5	0.49	0.8 ± 0.3
Kick off milker	4.0 (1.8, 8.6)	5.7 (2.8, 11.6)	0.5	0.47	0.7 ± 0.4
Entry ease ^2^					
1	68.0 (59.3, 75.6)	61.7 (52.4, 70.3)	1.2	0.27	1.3 ± 0.3
2	14.1 (9.3, 20.6)	21.9 (15.2, 30.4)	2.9	0.09	0.6 ± 0.2
>2	13.6 (8.3, 21.4)	13.3 (8.1, 21.0)	0.0	0.93	1.0 ± 0.3
Milking ease ^3^					
1	53.0 (45.1, 60.8)	40.2 (31.9, 49.1)	5.1	0.02	1.7 ± 0.4
2	35.0 (28.0, 42.8)	34.5 (26.6, 43.3)	0.0	0.92	1.0 ± 0.2
3	6.9 (3.7, 12.3)	13.8 (8.4, 21.8)	4.4	0.04	0.5 ± 0.2
>3	2.4 (0.9, 6.3)	8.3 (4.5, 15.0)	6.0	0.01	0.3 ± 0.2
Clinical mastitis	35.3 (19.4, 55.2)	23.7 (10.1, 46.3)	0.8	0.37	1.8 ± 1.1
Udder edema	6.3 (1.6, 22.1)	17.0 (5.9, 40.2)	1.8	0.18	0.3 ± 0.3

^1^ Chi-square statistic of likelihood ratio test (LRT). ^2^ 1 = willing; 2 = willing but hesitates; >2 = requires crowd gate prompt or requires handler to enter holding pen. ^3^ 1 = very calm; 2 = calm; 3 = restless; >3 = very restless or hostile.

**Table 4:** The probability (95% CI) of quarter IMI and pathogens isolated in milk within 36 h after calving in heifers receiving a weekly 1.0% iodine-based teat dip in the parlor 3 weeks prior to calving (trained) or no treatment (control).

	Treatment		Treatment Effect ^1^		Odds Ratio (OR) ± SE
Quarter IMI Indicators, % Probability	Train	Control	*χ* ^2^_(1)_	*p*-Value	Train vs. Control
Heifers, n	34	23			
Quarters evaluated, n	98	61			
Quarters contaminated, n	33	30			
Quarter infection level ^2^					
No growth	45.3 (34.4, 56.6)	34.8 (22.5, 49.6)	1.3	0.25	1.6 ± 0.6
Low	30.3 (21.3, 41.1)	23.7 (14.0, 37.2)	0.8	0.39	1.4 ± 0.5
Medium	7.3 (3.4, 15.0)	17.2 (8.4, 31.9)	3.1	0.08	0.4 ± 0.2
High	8.9 (4.0, 18.7)	16.7 (8.7, 29.5)	2.2	0.14	0.5 ± 0.2
Quarter pathogen isolate					
*Staphylococcus aureus*	4.2 (1.6, 10.7)	19.4 (10.0, 34.2)	8.1	<0.01	0.2 ± 0.1
CNS ^3^	34.0 (24.0, 45.6)	29.5 (18.7, 43.3)	0.3	0.58	1.2 ± 0.5
Other pathogen ^4^	12.4 (6.8, 21.4)	11.9 (5.4, 24.3)	0.0	0.93	1.1 ± 0.5

^1^ Chi-square statistic of likelihood ratio test (LRT). ^2^ None ≤ 1; low = 1–10; medium = 11–50; high ≥ 50 CFU/0.1 mL. ^3^ Coagulase-negative staphylococci. Includes *Staphylococcus chromogenes*, *Staphylococcus hominis*, *Staphylococcus sciuri* and non-*aureus Staphylococcus* spp. ^4^ Includes *Streptococcus dysgalactiae*, *Streptococcus uberis*, non-*agalactiae Streptococcus* spp., *Bacillus* spp., *Enterobacter cloacae*, *Enterococcus faecalis*, *Escherichia coli*, *Serratia* spp. and unidentified Gram-positive cocci.

## Data Availability

Data generated from this experiment are available on request from corresponding authors.
